# The development of narrative identity in the psychodynamic treatment of avoidant personality disorder: A case study

**DOI:** 10.3389/fpsyt.2023.1141768

**Published:** 2023-03-16

**Authors:** Ashley Frances Volodina Timberlake, Daniel Fesel

**Affiliations:** Department of Psychology, Goethe University Frankfurt, Frankfurt, Germany

**Keywords:** avoidant personality disorder, narrative identity, agency, communion fulfillment, coherence, short-term psychodynamic psychotherapy, case study

## Abstract

Avoidant personality disorder (AvPD) is characterized by feelings of shyness, inadequacy, and restraint in intimate relationships and has been associated with a disturbance in narrative identity, which is the internalized and evolving story of past, present, and future experiences. Study findings have indicated that an improvement in overall mental health through psychotherapy may increase narrative identity. However, there is a lack of studies incorporating not only the examination of narrative identity development before and after psychotherapy but also within psychotherapy sessions. This case study examined the development of narrative identity in short-term psychodynamic psychotherapy treatment of a patient with AvPD, using therapy transcripts and life narrative interviews before, after, and 6 months following treatment termination. Narrative identity development was assessed in terms of agency, communion fulfillment, and coherence. Results showed that the patient’s agency and coherence increased over the course of therapy, whereas communion fulfillment decreased. At the six-month follow-up, agency and communion fulfillment increased, whereas coherence remained stable. The results of this case study suggest that the patient’s sense of narrative agency and ability to narrate coherently improved after undergoing short-term psychodynamic therapy. The decrease of communion fulfillment during psychotherapy and later increase after termination suggests that the patient became more aware of conflictual patterns in their relationships, therefore realizing that their wishes and desires were not being fulfilled in their current relationships. This case study displays the possible impact short-term psychodynamic therapy may have by helping patients with AvPD develop a narrative identity.

## 1. Introduction

Previous narrative research has identified disturbances in narrative identity of individuals with personality disorders (PD) (1–3). Whilst most studies have mainly focused on general features of PDs or specifically on borderline personality disorder (BPD) (3), avoidant personality disorder (AvPD) has been largely neglected, despite its high prevalence, mortality rate, and degree of subjective impairment (4). Furthermore, there remains an ongoing debate on which elements of narrative identity may change through psychotherapy. Moreover, there is a lack of research incorporating not only the examination of changes in narrative identity before and after psychotherapy but also within psychotherapy sessions. Finally, most studies also do not to include a follow-up examination to determine how long-lasting changes in narrative identity are after treatment termination.

The present case study aims at investigating the development of the narrative identity of a patient with AvPD. For this examination, life stories before, after, and 6 months following short-term psychodynamic treatment and narratives within psychotherapy sessions from therapy transcripts are utilized to identify changes in agency, communion fulfillment, and coherence. We will first illustrate the importance of examining AvPD, then introduce the concept of narrative identity in terms of agency, communion fulfillment and coherence, and finally outline past research findings regarding disturbances in narrative identity of patients with personality disorders as well as changes in narrative identity through psychotherapy.

Avoidant personality disorder is a severe disorder characterized by social inhibition, hypersensitivity to negative evaluation by others, and feelings of inadequacy (5). The community prevalence rate for AvPD, including men and women, lies at 3.3% (6), while it is estimated that AvPD as a comorbid disorder is diagnosed up to 14.7% in psychiatric outpatients (4). However, most people diagnosed with AvPD usually receive treatment for depression, anxiety, or psychosis, and it may be the case that inpatient treatment may even overlook the symptoms of AvPD in clinical settings. Many cases of AvPD treatment focus on treating social fears (7). Furthermore, AvPD has also been linked to a high rate of morbidity, making it of high clinical and research interest (6). Compared to other personality disorders (PD), it was found that AvPD had the highest rate of impairment in daily functioning (8). However, despite its high prevalence and subjective impairment, AvPD is severely understudied, especially in the research field of narrative identity (3).

*Narrative identity* is defined as the internalized and further evolving story of the self, which an individual constructs through self-reflection and meaning-making of their own life e.g., (9–11). As narrative identity has been related to mental health and psychological well-being (12), various research has been conducted on examining individual differences in personal stories e.g., (13–15). A variety of elements characterizes narrative identity, among others, motivational elements such as agency and communion (16, 17), as well as structural elements such as coherence (14, 18, 19).

According to McAdams et al. (20), agency is considered one of the central thematic clusters in personal narratives and life stories. The theme of agency represents the individual’s perception of their achievements, mastery, autonomy, and ability to influence circumstances and the course of their own life; thus, it is viewed as a central element that provides information to what degree an individual experiences a sense of meaning and purpose.

Communion is considered the second major motivational thematic cluster, next to agency. Communion represents the degree to which an individual’s narrative portrays experiences of interpersonal connection and harmony with others (21). Narratives high in communion emphasize motivation for love, attachment, belongingness, and friendship (20) and have been related to higher well-being in previous studies (22). Communion fulfillment refers to whether or not communal needs and desires are fulfilled (1).

Furthermore, the ability to deliver a highly coherent narrative of past experiences is associated with how well an experience has been dealt with (23–25). Thus, coherently structured stories are associated with having worked through and moved on from an experience (26). Furthermore, narratives regarded as organized, detailed, and elaborative have previously been associated with greater well-being and fewer psychiatric disturbances (1, 14, 27).

Studies investigating changes in narratives have shown that narratives naturally change throughout the lifespan (28). Furthermore, significant others, such as family members, may also co-author narratives and influence the development of narrative identity (29–31). Despite narrative identity changing over time, an intense examination of narratives regarding past experiences through psychotherapy may also lead to changes in narrative identity: A therapist may be perceived as a “special” co-author, who influences the change on narratives more intensively with the use of different technical means (32). For example, theoretical assumptions from psychoanalytic ego psychology suggest, that neurotic defense-related distortions may lead to narratives being incomprehensible, implausible, or contradictory (33). The therapist’s work involves clarifying unclear details and implications of narratives with the patient as well as confronting contradictions and missing or unclear motives in order to reconstruct a more coherent story. According to Schafer (33) and Argelander (34), unconscious conflicts evolve around desires; therefore, conflictual motives are omitted and hidden in the autobiographical narratives of patients. This may result in a construction of the life story in which the patient is portrayed as not being agentic or responsible but instead views him-or herself as a passive victim of circumstances. Therefore, psychodynamic work aims at helping the patient create a life story in which they perceive themselves as agentic and responsible for their lives (32).

Many studies have illustrated the role of disturbances in narrative identity within personality disorders. While previous research has focused on borderline personality disorder (BPD) or general PD features, little attention has been drawn to AvPD.

When comparing the theme of agency in the life story interviews of BPD patients with those of matched community control participants, patients with BPD had low agency scores which were expressed through passivity, low mastery, and victimization (1, 35). A qualitative study examining BPD patients’ life stories revealed themes of low self-worth and struggling with gaining control over emotions, behavior, and purpose in life (36). Furthermore, Gilbert et al. (37) examined life story interviews with people who had manifested features of PD. Findings showed that many expressed a sense of powerlessness and inability to initiate change, indicating an association between PD and low agency.

Previous studies have also found that individuals with a BPD diagnosis or PD features did not indicate less need for communion. Instead, communal themes were described as less fulfilling, illustrating low communion fulfillment. During life story interviews, patients reported experiencing complex family dynamics, neglect, and feelings of being different or distant from others. Many individuals also reported struggling with developing trusting relationships (1, 37, 38).

Regarding narrative coherence, previous research suggests that the life stories of people with BPD features are lower in overall coherence (1) compared to narratives of matched controls. Similarly, the findings of Lind et al. (39) suggest an association between lower narrative coherence in life stories and elevated features BPD among inpatient adolescents. Life stories of individuals struggling with BPD have been characterized by less integration of the current self and less meaning-making, resulting in overall lower narrative coherence (40).

However, most studies mainly focus on BPD or PD features (3), and there is a lack of research conducted on examining the narrative identity disturbances of participants with AvPD. A qualitative study (41) examining narratives of everyday life struggles told by 15 AvPD patients found that thwarted themes of agency and communion fulfillment characterized narratives. Individuals described a fleeting sense of self in which they felt they were not in control of their lives and were constantly doubting themselves and their ability to initiate change. Furthermore, Individuals struggled with a longing for a connection with others while also dreading interpersonal relationships and were inclined to isolate themselves instead of fulfilling their communal needs.

A case point delivered by Lind et al. (42) examined narratives of the life story interview of a patient with AvPD and comorbid BPD. These narratives were also characterized by thwarted agency. The patient described himself as unable to reach his goals and portrayed himself as a passive victim of external events and circumstances. Furthermore, narratives included many communal themes but were associated with low levels of communion fulfillment, as the patient’s longing for relationships had been repeatedly dissatisfied. It is suggested that these struggles portrayed in the patient’s narratives may be treated with a narrative-repair-focused treatment (42).

Previous findings suggest that individuals who undergo psychotherapeutic treatment display changes in their life stories and narratives before and after as well as over the course of treatment. However, there remains an ongoing debate which elements of narrative identity may change through psychotherapy. Furthermore, studies have used differing forms of methods to examine these changes. Moreover, previous research has mainly focused on patients with BPD, and to our knowledge no studies have yet been conducted with patients suffering from AvPD only.

A study conducted by Adler (22) examined personal narratives of 47 adults, which were written prior to the beginning and after every psychotherapy session over a period of 12 weeks. Participants were concurrently measured for mental health. Narratives were examined for themes of agency and coherence to capture narrative identity in terms of purpose and unity. Results indicated that agency increased over the course of psychotherapy, whereas coherence did not. Increases in agency were significantly related to overall improvements in participants’ mental health. Furthermore, findings also revealed that changes in the theme of agency occurred prior to improvements in overall mental health. However, this study did not examine psychotherapy transcripts but instead asked participants to write narratives evaluating their current psychotherapy session. These narratives may not have been narrative in nature but instead, portray a more filtered form of narration. The missing increase in coherence was attributed to the fact that the narratives examined in this study regarded experiences of the present day. In contrast, most studies have examined coherence in narratives of retrospective accounts. It was therefore suggested that an increase in coherence occurred over time and was not due to psychotherapy. It was also pointed out that the overall course of coherence, whether that may be an increase or a decrease, could be linked to individual differences. However, narratives were written instead of verbally told. The act of writing narratives itself has been previously linked to improvements in psychological well-being (25). Therefore, these narratives do not truly represent the effects of psychotherapy on narrative identity.

In contrast, other studies examining changes in narrative coherence through psychotherapy treatment have found patients with posttraumatic stress disorder and schizophrenia to report more coherent trauma narratives (43) or life stories (44) after psychotherapy. Furthermore, the findings of a case study by Lysaker et al. (45) also demonstrated that the told narratives of a schizophrenic patient within psychotherapy sessions became more coherent over the course of treatment.

Research investigating changes in narrative identity of patients with PD features through psychotherapy has been scarce and has merely focused on BPD. Lind et al. (35) investigated whether a 12-month psychotherapy treatment would affect how BPD patients narrated their personal life stories and that of their parents during life story interviews. After treatment termination, BPD patients’ life stories had increased significantly in themes of agency, whereas no significant changes were found in terms of communion. However, no follow-up examination was conducted in this study. Regarding narrative coherence, Levy et al. (46) found that narratives derived from an Adult Attachment Interview of BPD patients were significantly more coherent after 12 months of transference-focused treatment.

Despite an abundance of research on narrative identity and its interplay with personality psychopathology and psychotherapy, there remains a lack of agreement on whether psychotherapy may contribute to an increase in narrative coherence and communion fulfillment. In addition, AvPD has been largely neglected in narrative identity research, especially regarding disturbances in narrative coherence and changes in narrative identity due to psychotherapy treatment. Furthermore, there is a lack of research incorporating not only the examination of changes in narrative identity before and after psychotherapy but also within psychotherapy sessions.

Finally, many studies have not conducted a follow-up examination to determine how long-lasting changes in narrative identity are after psychotherapy. Thus, this qualitative case study aims to examine the development and change of an AvPD patient’s narrative identity within psychotherapy sessions as well as before, after, and 6 months following short-term psychodynamic psychotherapy (STPP). The study inquires whether narrative identity in psychotherapy sessions and the patient’s life story will increase in terms of agency, communion fulfillment, and coherence after undergoing STPP. Moreover, the present study investigates whether changes remain stable in a 6-month follow-up.

We hypothesized that the patient’s narratives within psychotherapy sessions will increase over the course of treatment in agency (H1), communion fulfillment (H2), and coherence (H3). We also expected that the life stories of the patient after STPP and 6 months following treatment termination will be higher in agency (H4), communion fulfillment (H5), and coherence (H6) when compared to the life story before treatment.

## 2. Methods

### 2.1. The patient

The patient in question was 28 years old at the beginning of therapy, seeking treatment because of issues at work and also due to excessively struggling with a previously failed relationship. The patient had recently started her first job after graduating from university. She appeared somewhat shy and anxious in contact but reported openly and visibly motivated about her problems and biography. She reported not being successful at setting boundaries or demanding things for herself. In her workplace, she had recently burst into tears several times due to feeling overwhelmed. She also mentioned that she often suffered from feelings of guilt. The patient had no previous psychotherapeutic experience.

### 2.2. Procedure

Data used in this study were derived from a pilot study conducted for psychotherapy research by doctoral student Daniel Fesel. The original study was designed to collect data on a wide spectrum of psychological disorders. Two patients took part in the pilot study: The first patient was an abstinent alcoholic who suffered from panic attacks and nightmares. The second patient was diagnosed with AvPD and is the patient of this case study. This particular case study was selected for further examination due to the patient’s diagnosis and the lack of research on changes in narrative identity of individuals with AvPD. The present study will only use data regarding one of the cases from the pilot study, namely all life story narratives collected at interview time points, therapy transcripts, and questionnaire data. All assessments were conducted in German, including narrative interviews, therapy sessions, and questionnaires.

After seeking treatment at a psychodynamic outpatient clinic, the patient received several consultation sessions. In the third session, the psychotherapist offered the patient a STPP treatment consisting of 24 sessions on the condition that she participates in the psychotherapy study, which was approved by the JGU’s Ethics Committee in December 2017. In the fourth session, the patient expressed her willingness to participate in the study and signed a written consent form. After three more probationary sessions, the psychotherapy application was approved by the patient’s health insurance. The patient had therefore received a total of seven probatory sessions. During this period, the patient participated in the pre-interview (T1), which included a diagnostical examination, a test battery, and a narrative interview. The diagnostical examination consisted of the Structural Clinical Interview for the DSM (SCID-I and SCID-II) (47) which an external diagnostician conducted on two dates. In the narrative interview, which a separate diagnostician conducted, the patient was asked to write seven life events on index cards and then to tell her life story without interruption, taking these seven events into account. The instruction was the same for all interviews. The patient could, therefore, include other or the same events in her life story during the follow-up interviews. In addition, the patient filled out a test battery during the same period, which included questionnaires examining depressive symptoms (BDI-II) (48), psychological distress and psychiatric disorders (BSI) (49), personality dysfunction (OPD-SQS) (50), and difficulties in interpersonal relationships (IIP-32) (51). Per the SCID-I interview, the patient was diagnosed with generalized anxiety disorder and somatization disorder. According to the SCID-II, she was diagnosed with an AvPD.

At the beginning of therapy, the male therapist had a license to practice psychodynamic psychotherapy, which he had received at a recognized psychoanalytic training institute. Including the practical activities during the training, the therapist had 4.5 years of practical experience at the time of the study’s baseline.

The 24 sessions of the STPP took place once a week in a seated setting with some interruptions, such as holidays. Contemporary psychodynamic psychotherapy is based on therapeutic approaches derived from traditional psychoanalytic theory and therapy (52). However, this form of psychotherapy focuses less on the personal past and more on present-day conflicts. Moreover, psychodynamic psychotherapy explores the patient’s unconscious conflicts, past relationship experiences, and how their personality and previous coping mechanisms have contributed to the manifestation of their symptoms. A focal aim is identified and central conflicts that are present in the here-and-now and are associated with current manifested symptoms are worked through (52, 53).

After every fourth session, the Outcome Questionnaire 45 (OQ-45) (54) measuring the patient’s symptom severity and, therefore, the progress of therapy, was filled out by the patient. The duration from the first to the last therapy session resulted in 40 weeks. All probatory and therapy sessions were audio-recorded; however, due to missing or faulty recordings, the 18th and the 24th therapy sessions were unavailable as audio recordings.

Two weeks after the last therapy session, the second narrative interview, the post-interview (T2), took place, in which the patient filled out the questionnaire battery and took part in the narrative interview. The pre-and post-interview took place 11 months apart. Six months after the termination of psychotherapy, the patient took part in the last narrative interview, the follow-up-interview (T3), with an analogous structure to the pre-and post-interview, and for the final time, filled out the test battery. Each of the three interviews was conducted by different diagnosticians. All three interviewers and diagnosticians conducted blind assessments. They were not aware of any information established in other interviews, within psychotherapy treatment, and were also not aware of the hypotheses of the study.

### 2.3. Case illustration

#### 2.3.1. Interview test battery

All material descriptions for the clinical assessment used during the interview test battery are listed in Table 1.

**Table 1:** Descriptions for the clinical assessments within the interview test battery.

Materials	Description
Becks depression inventory (BDI-II)	The becks depression inventory (BDI-II) (48) includes 21 items and assesses the severity of depression symptoms on a 4-point scale ranging from 0 to 3. This tool is not used for diagnostic purposes but instead serves as a screening tool. Higher scores indicate more severe depressive symptoms. The self-questionnaire has multiple cut-off ranges: 0–13 is considered the range for none to minimal symptoms of depression; 14–19 stands for mild depression; 20–28 for moderate depression; and 29–63 for severe depression. The questionnaire has a high degree of internal consistency (*a* = 0.84) (55)
Brief symptom inventory (BSI)	The brief symptom inventory (BSI) (49) includes 53 items and is a self-report measurement used for the screening of psychological distress and psychiatric disorders and is used to monitor treatment progress and assess treatment outcome. Level of distress during the past week is assessed on a five-point Likert scale (ranging from 0 = “not at all” to 4 = “extremely”). Nine primary symptom dimensions are measured: Somatization, Obsession-compulsion, Depression, Anxiety, Interpersonal sensitivity, Hostility, Phobic anxiety, Paranoid ideation, and Psychoticism. In addition, a Global Severity Index based on all 53 items provides an overall score across all nine domains. All scores are transformed into T-scores. T-Scores equal or above 63 are viewed as clinically relevant. The Global Severity Index (*a* = 0.97) has an excellent degree of internal consistency. The dimensions Somatization (*a* = 0.85), Obsession-compulsion (*a* = 0.84), Interpersonal sensitivity (*a* = 0.85), Depression (*a* = 0.88), Anxiety (*a* = 0.86), Hostility (*a* = 0.81), and Paranoid ideation (*a* = 0.81) have a high degree of internal consistency. Furthermore, the dimensions of Phobic anxiety (*a* = 0.78) and Psychoticism (*a* = 0.73) have a satisfactory degree of internal consistency (56)
OPD-structure questionnaire short version (OPD-SQS)	The OPD-structure questionnaire short version (OPD-SQS) (50) is a 12-item screening tool for personality dysfunction, which is used in planning or assessing change throughout psychotherapy on a five-point Likert scale (ranging from 0 = “fully disagree” to 4 = “fully agree”). The questionnaire includes three personality domains: self-perception, relationship model, and contact design. The domain of self-perception entails to what degree the self is equipped with structural skills and emotion regulation. The domain relationship model encompasses to what degree representations of past relationships, associated with expectations for new relationships, are dysfunctional. The domain contact design portrays skills in interpersonal contact with respect to self-insecurity. Higher scores illustrate a higher level of personality dysfunction. The questionnaire has a high degree of internal consistency (*a* = 0.88) (50)
Inventory of interpersonal problems (IIP-32)	The short form of the inventory of interpersonal problems (IIP-32) (51) includes 32 items and is a self-assessment tool that captures difficulties and sources of distress within interpersonal relationships. It is used to assess psychotherapy progress on a five-point Likert scale (ranging from 0 = “not at all” to 4 = “extremely”). Items are phrased in two ways: difficulties are either experienced as things individuals do “too much” of or things individuals find “too hard”. Eight primary interpersonal behavior domains are assessed: Domineering/Controlling, Vindictive/Self-Centered, Cold/Distant, Socially Avoidant/Inhibited, Nonassertive, Overly Accommodating/ Exploitable, Self-Sacrificing/Overly Nurturant, and Intrusive/Needy. A sum score based on all 32 items provides an overall score across all eight domains. All scores are transformed into stanine points. Stanine points above six are viewed as clinically relevant. The IIP-32 global score (*a* = 0.93) and the domain Vindictive/Self-Centered (*a* = 0.92) have an excellent degree of internal consistency. The domains Cold/Distant (*a* = 0.84), Socially Avoidant/Inhibited (*a* = 0.89), Nonassertive (*a* = 0.86), and Self-Sacrificing/Overly Nurturant (*a* = 0.80) have a high degree of internal consistency. Furthermore, the domains Domineering/Controlling (*a* = 0.73), Overly Accommodating/Exploitable (*a* = 0.78), and Intrusive/Needy (*a* = 0.76) have a satisfactory degree of internal consistency (57)
Outcome questionnaire 45 (OQ-45)	The outcome questionnaire 45 (OQ-45) (54) is a 45-item self-report questionnaire measuring overall distress and used to assess psychotherapy progress in adults on a five-point Likert scale (ranging from 0 = “never” to 4 = “almost always”). It is not used for diagnostic purposes but instead as a screening tool. Overall distress is measured in three domains: Symptom distress, interpersonal relationships, and social role. The domain symptom distress measures the degree of subjective distress from symptoms. The domain of interpersonal relationships measures the degree of dysfunctionality in interpersonal relationships. The domain social role measures the degree of dysfunctionality at work and in other social roles. All three domains are summed up to an overall distress score. Higher scores indicate a higher degree of disturbance and distress for an individual. The questionnaire has an excellent degree of internal consistency (*a* = 0.94) (58)

##### 2.3.1.1. BDI-II

**Figure 1:**
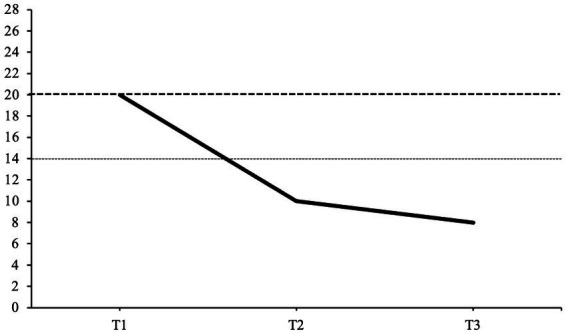
Back’s depression inventory-II scores at each interview time point. This graph displays the patient’s BDI-II scores at each interview time point. T1, pre-interview; T2, post-interview; T3, follow-up interview; 0–13, clinically unremarkable; 14–19, mild depression; 20–28, moderate depression; 29–63, severe depression. The scale initially ranges from 0 to 63. Dashed lines represent the cut-off thresholds for mild and moderate depression.

The patient’s depressive symptoms were assessed with the BDI-II (48). The patient’s BDI-II scores at each interview time point are illustrated in Figure 1. Before starting STPP, the patient scored 20 on the BDI-II in the pre-interview, reaching the threshold marking moderate depressive symptomatic. The patient’s BDI-II score at treatment termination had decreased to a score of 10, meaning the amount of reported depressive symptoms was clinically unremarkable. At the follow-up interview, the patient’s BDI-II score had further decreased to 8, remaining clinically unremarkable.

##### 2.3.1.2. BSI

The patient’s psychological distress and psychiatric disorder symptoms were assessed with the BSI (49). All BSI scores at all interview time points are presented in Table 2. Before the beginning of therapy, the patient had an overall global score of 71, which is considered a clinically relevant level of psychological distress. The patient had scored significantly high on all symptom domains apart from Somatization. After treatment termination, the patient’s BSI score remained the same; however, only the following symptom domains had reached the cut-off score: Obsession-compulsion, Anxiety, Hostility, Phobic anxiety, and Paranoid ideation. At the follow-up interview, the patient’s BSI score had decreased to an overall global score of 60, illustrating a non-clinically relevant level of psychological distress. Hostility and Phobic anxiety were the only two domains in which the patient’s score remained clinically significant.

**Table 2:** BSI scores at each interview time point.

	Interview time point		
	T1	T2	T3
Somatization	56	59	52
Obsession-compulsion	**64**	**66**	62
Interpersonal sensitivity	**65**	61	52
Depression	**68**	49	57
Anxiety	**64**	**69**	59
Hostility	**66**	**72**	63
Phobic anxiety	**73**	**70**	**70**
Paranoid ideation	**65**	**69**	54
Psychoticism	**70**	59	54
Global Score Index	**71**	**71**	60

T1, pre-interview; T2, post-interview; T3, follow-up interview. All scores are presented as T-scores. Scores equal or greater 63 are in bold and considered clinically relevant.

##### 2.3.1.3. OPD-SQS

**Figure 2:**
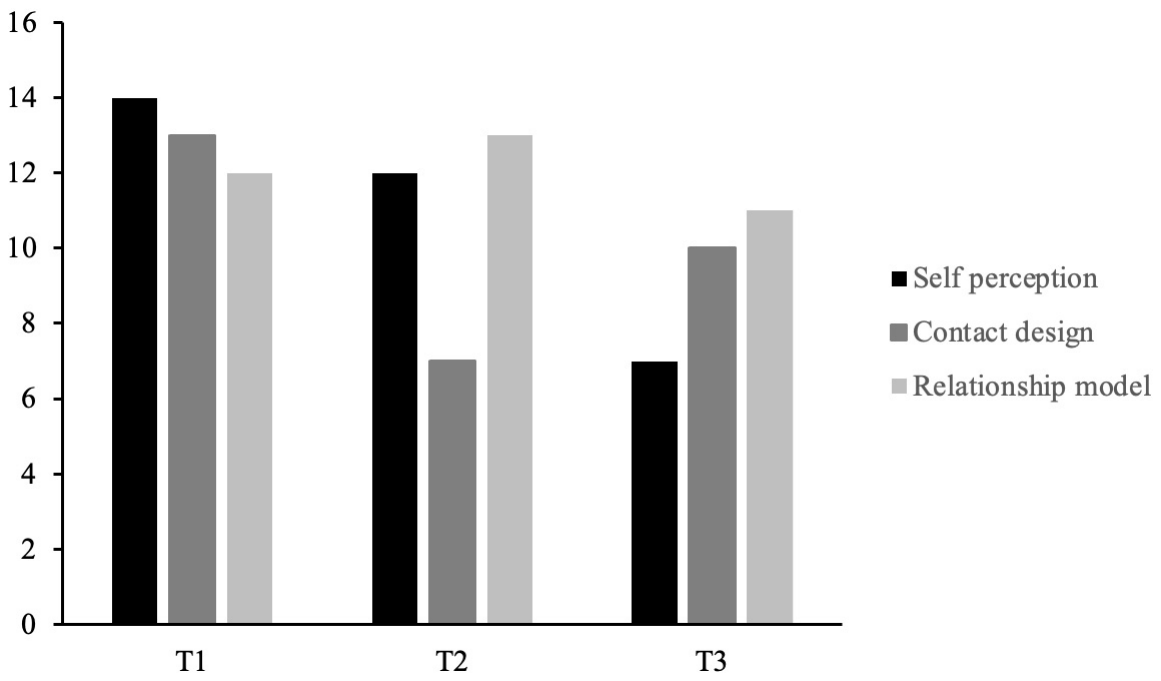
OPD-SQS domain scores at each interview time point. This graph illustrates the patient’s OPD-SQS scores for the domains self-perception, contact design and relationship model at each interview time point. T1, pre-interview; T2, post-interview; T3, follow-up interview.

The patient’s personality dysfunction was assessed with the OPD-SQS (50), with which the following domains are measured: self-perception, relationship model and contact design. All OPD-SQS domain scores for each interview time point are illustrated in Figure 2. Before starting treatment, the patient scored high on dysfunctional self-perception with a score of 14, contact design with a score of 13, and moderately high on relationship model with a score of 12. After treatment termination, the patient’s self-perception score had decreased to a moderately high score of 12. In contrast, the patient’s contact design score post-treatment had largely decreased to 7. The relationship model score had increased to a score of 13. At the follow-up interview, the patient’s self-perception score had largely decreased to a score of 7. In contrast, contact design had increased to a moderate score of 10, and the relationship model score had decreased to a moderate score of 11.

##### 2.3.1.4. IIP-32.

The patient’s difficulties in interpersonal relationships were assessed with the IIP-32 (51). All IIP-32 scores at all interview time points are presented in Table 3. Before the beginning of therapy. The patient had an overall score of 9, which is considered a clinically relevant level of distress within interpersonal relationships. The patient had scores significantly high in all interpersonal domains. After treatment termination, the patient’s overall score decreased to 7, remaining in the clinically relevant range; however, only the following interpersonal domains had reached the cut-off score: Nonassertive, Overly Accommodating/Exploitable, and Self-Sacrificing/Overly Nurturant. At the follow-up interview, the patient’s IIP-32 score remained the same. The following interpersonal domains had reached the cut-off score: Socially Avoidant/Inhibited, Overly Accommodating/Exploitable, and Self-Sacrificing/Overly Nurturant.

**Table 3:** IIP-32 scores at each interview time point.

	Interview time point		
	T1	T2	T3
Domineering/controlling	**8**	6	6
Vindictive/self-centered	**7**	4	5
Cold/distant	**7**	5	5
Socially avoidant/inhibited	**9**	6	**8**
Nonassertive	**9**	**8**	6
Overly accommodating/exploitable	**8**	**8**	**8**
Self-sacrificing/overly nurturant	**7**	**8**	**9**
Intrusive/needy	**7**	**6**	6
Overall score	**9**	**7**	**7**

T1, pre-interview; T2, post-interview; T3, follow-up interview. All scores are presented as Stanine-scores. Scores greater 6 are in bold and considered clinically relevant.

##### 2.3.1.5. OQ-45

**Figure 3:**
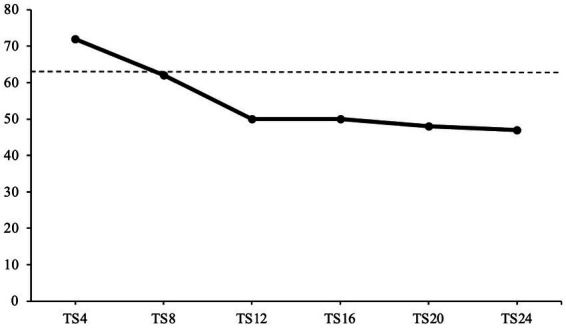
OQ-45 scores over the course of treatment. This graph displays the patient’s OQ-45 scores over the course of treatment. TS, therapy session. Dashed line represents the cut-off threshold at score 63.

The patient’s symptom severity was measured with the OQ-45 (54). Overall distress is measured in three domains: Symptom distress, interpersonal relationships, and social role. All three domains are summed up to an overall distress score. All OQ-45 scores throughout therapy are illustrated in Figure 3. At the beginning of treatment, the patient’s overall distress is significantly high, with a score of 72 after the fourth therapy session. After the eighth therapy session, the patient’s distress levels decrease just below the cut-off threshold with a score of 62. During the middle period of therapy, the patient’s distress levels remain stable and clinically insignificant, with a score of 50 in sessions 12 and 16. Towards the end of STPP, the patient’s scores decrease slightly to a score of 48 in session 20 and a score of 47 in session 24, remaining clinically insignificant.

#### 2.3.2. Psychodynamics

Before discussing the patient’s psychodynamics in the research group, all interviews and therapy sessions were transcribed verbatim by research assistants following the basic version of GAT transcription rules (59). All identifying information (e.g., names, occupation) were replaced by pseudonyms.

To establish the patient’s psychodynamics, the research group including two psychology master’s students and a doctoral student with psychotherapeutic experience met weekly for eight discussion meetings of one and a half hours. During these weekly discussions, four therapy transcripts were read, and the content was discussed, with the goal of understanding the course of the patient’s psychotherapy and carving out the patient’s psychodynamics. In addition to the therapy transcripts, the therapist presented the group with a written summary of the course of therapy and the psychodynamic impressions of the patient in form of an epicrisis. The discussion group took all data including all interviews and questionnaires into account when establishing the patient’s psychodynamics.

During the pre-interview and probatory sessions, the patient expressed the wish to become better at setting boundaries and being able to say no to others. She reported difficulties in asserting her needs and interests toward others. When not fulfilling the needs of people in her life, the patient experienced inexplicable feelings of guilt. Furthermore, the patient described herself as a person who pays much attention to how others might think about her, therefore trying to please others while not considering what she might want instead. The patient explained that she had never learned to argue or stand up for herself, addressing this as her main goal for her upcoming psychotherapy treatment.

Throughout psychotherapy and during further discussion in the research group, the following psychodynamics were established: The patient suffered early developmental deficits resulting from her birth complications and following necessary treatment, as well as growing up under the strong influence of her anxious parents. The patient reported experiencing the impression of a stigma or deficit since childhood: the feeling of progressing slower than others, which resulted in persistent feelings of shame. This affected her social skills, making her quiet, shy, and inhibited. During treatment, the patient displayed insecurity about her identity and low self-esteem. These insecurities were expressed, for example, in her shyness towards conflict and a rather underdeveloped integration of aggressive impulses. When experiencing anger in conflictual situations at work, she helplessly bursts into tears and feels ashamed afterward. The patient strongly tended to feel guilty but behaved accusingly and passive-aggressively towards others. She feared that other people would react harshly and punitively to her mistakes. She perceived herself in a negatively distorted way and was afraid of appearing too aggressive when standing up for herself. The patient also exhibited an over-demanding self-ideal, which was expressed in an exaggerated urge to solve the problems of others and feeling guilty when taking time off from work. Due to the patient being insecure regarding her identity and suffering from low self-worth, the patient remained passive and submissive in her relationships, for example, by putting herself in a dependent position in her failed relationship and allowing herself to be dominated or by almost always agreeing with the therapist. Throughout therapy, she revealed a desire for a stable romantic relationship in which all facets of her personality are accepted, especially those she devalues.

### 2.4. Narratives

All narratives within the therapy transcripts were identified by a previous master’s student (60) who had identified all narratives in the therapy transcript based on an unpublished manual (61). Narratives were identified based on Labov’s definition, namely that a narrative must contain at least two narratives clauses separated by a temporal junction. Narrative clauses consist of a report of events reflecting the order in which these events took place. Therefore, narrative clauses report an event with a definable beginning and end. Furthermore, the order in which narrative clauses are told may not be altered without changing the meaning of the reported event (62).

#### 2.4.1. Narrative coding

Further narrative coding was to be conducted for the life stories of all three interviews and narratives from the psychotherapy transcripts. Before randomly choosing narratives for further coding, all transcripts were read and discussed in a research group to understand the patient’s issues and psychodynamics better. As the patient’s struggles primarily evolved around interpersonal relationships and romantic relationships were a key motive throughout the patient’s STPP, all narratives revolving around romantic relationships were identified in the listing provided by Kinder (60). Five sessions in total were chosen, in which two randomly chosen narratives in which the patient narrated an experience in romantic interpersonal relationships. This resulted in a coding of 10 narratives from the therapy transcripts: four narratives from the first third of therapy (from sessions two and five), two narratives from the second third of therapy (from session 14), and finally, four narratives from the last third of therapy (from sessions 17 and 23). For further coding, all 10 narratives from the therapy transcript and the life story narratives were propositioned in main-and sub-clauses.

For all narratives, coding was conducted by theme: every narrative was coded for agency, then communion fulfillment, and finally for all coherence dimensions. This meant that each narrative received six separate readings throughout the coding process. An average score for each of the three narrative identity dimensions was calculated for each narrative and then for each session. Concerning the life story, each of the seven individual life story events was coded separately, and a mean narrative identity score was calculated for the whole life story. The following utilized coding schemes were used due to previous researchers achieving high inter-rater reliability (1, 22, 35).

A master’s student in psychology served as a master coder and coded all interview and therapy session narratives. The master coder was not blinded during coding since the entire working group had used a bottom-up approach by first reading all transcripts, working out all central themes, and finally developing the hypothesis before starting the coding process. In a separate step, a PhD-student recoded all narratives as a reliability coder, making use of the master coder’s prepared coding segments (63).

##### 2.4.1.1. Agency

The agency coding system by Adler et al. (64) is used to measure agency in narratives with the help of a five-point Likert scale ranging from 0 to 4. Higher scores indicate a higher degree of agency. A narrative in which the protagonist is completely powerless, at the mercy of circumstances, and all action is motivated by external powers is scored with 0. A narrative that portrays the protagonist as largely at the mercy of circumstances and in which the control of the plot is primarily in the hands of external powers is rated with 1. A narrative in which the protagonist is neither entirely in control nor entirely at the mercy of circumstances receives a score of 2. A narrative in which the protagonist is able to affect actions, initiate change, or has some degree of control over circumstances is rated with 3. These narratives may or may not include a portrayal of previous agentic struggles. A narrative in which the protagonist reports having struggled to overcome an agency-threatening experience and describes themselves as empowered or victorious is rated with 4. These narratives include themes of self-insight, gaining control over a situation, or experiencing an increase in power.

For both life narratives (*ICC* = 0.94) and therapy session narratives (*ICC* = 0.86), excellent inter-rater reliabilities were achieved (65).

##### 2.4.2.1. Communion fulfillment

Adler et al. (1) communion fulfillment coding system measures communion fulfillment in personal narratives and consists of a 3-point scale ranging from 0 to 2. The coding system of communion fulfillment goes further to examine whether there is an absence or presence of communion motivation. It instead also examines to what degree the protagonist is successful in satisfying their motivational needs and desires in interpersonal relationships. Since narratives chosen from the psychotherapy transcripts evolved around interpersonal relationships, rating the presence or absence of communal motivations was unnecessary. However, in the case of the life story narratives, narratives were rated with 0 for the absence and 1 for the presence of communal motivations. In case no communion was recorded, the narrative did not receive a score for communion fulfillment. Narratives in which there was no indication of having one’s communion needs met were scored with 0. Narratives in which communion needs were being met to some degree were scored with 1. Narratives in which the protagonist’s communion needs were met to a high degree were scored with 2.

For both, life narratives (*ICC* = 0.94) and therapy session narratives (*ICC* = 0.84), excellent inter-rater reliabilities were achieved.

##### 2.4.2.2. Coherence

The narrative coherence coding system (14) is a rating scale used to measure coherence in life-story accounts. This coding system has been used before to assess the coherence of psychotherapy stories (66). Coherence is conceptualized along four dimensions: orientation, structure, affect, and integration. Each dimension is rated along a 4-point scale ranging from 0 to 3. Higher scores indicate a higher degree of coherence. All four dimensions were summed up to an average coherence score.

**Figure 4:**
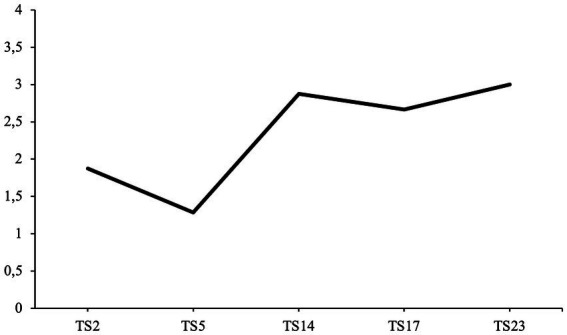
Average agency scores over the course of treatment. TS, therapy session.

*Orientation* is defined as the extent to which the narrator specifies background information to understand the story’s context. This includes introducing main characters, locations, and actions and situating the narrative in a specific temporal, social, and personal context. *Structure* is defined as the level of temporal sequencing within the story (Figure 4). A narrative must therefore include one of the following: an initiating event, an internal response to the event, an attempt, or a consequence. Highly structured narratives follow a temporal sequence of goal-oriented action and present a logical flow of scenes. *Affect* is defined as the extent to which a narrative includes expressive emotion and makes an evaluative point by using affective language. Through the use of expressive emotion, the narrator underlines the importance of the experience being recounted. *Integration* is the degree to which the narrator links the event to larger life themes and meaning. A highly integrated narrative relates the events to the narrator’s self-identity or previous autobiographical experiences.

For all coherence scales in the life narratives a good or excellent inter-rater reliability was achieved (orientation *ICC* = 0.66, structure *ICC* = 0.76, affect *ICC* = 0.93, integration *ICC* = 0.89) while in the therapy session narratives all scales reached the classification of an excellent inter-rater reliabilities (orientation *ICC* = 0.89, structure *ICC* = 0.78, affect *ICC* = 0.96, integration *ICC* = 0.85).

## 3. Results

### 3.1. Psychotherapy narratives

#### 3.1.1. Agency

**Figure 5:**
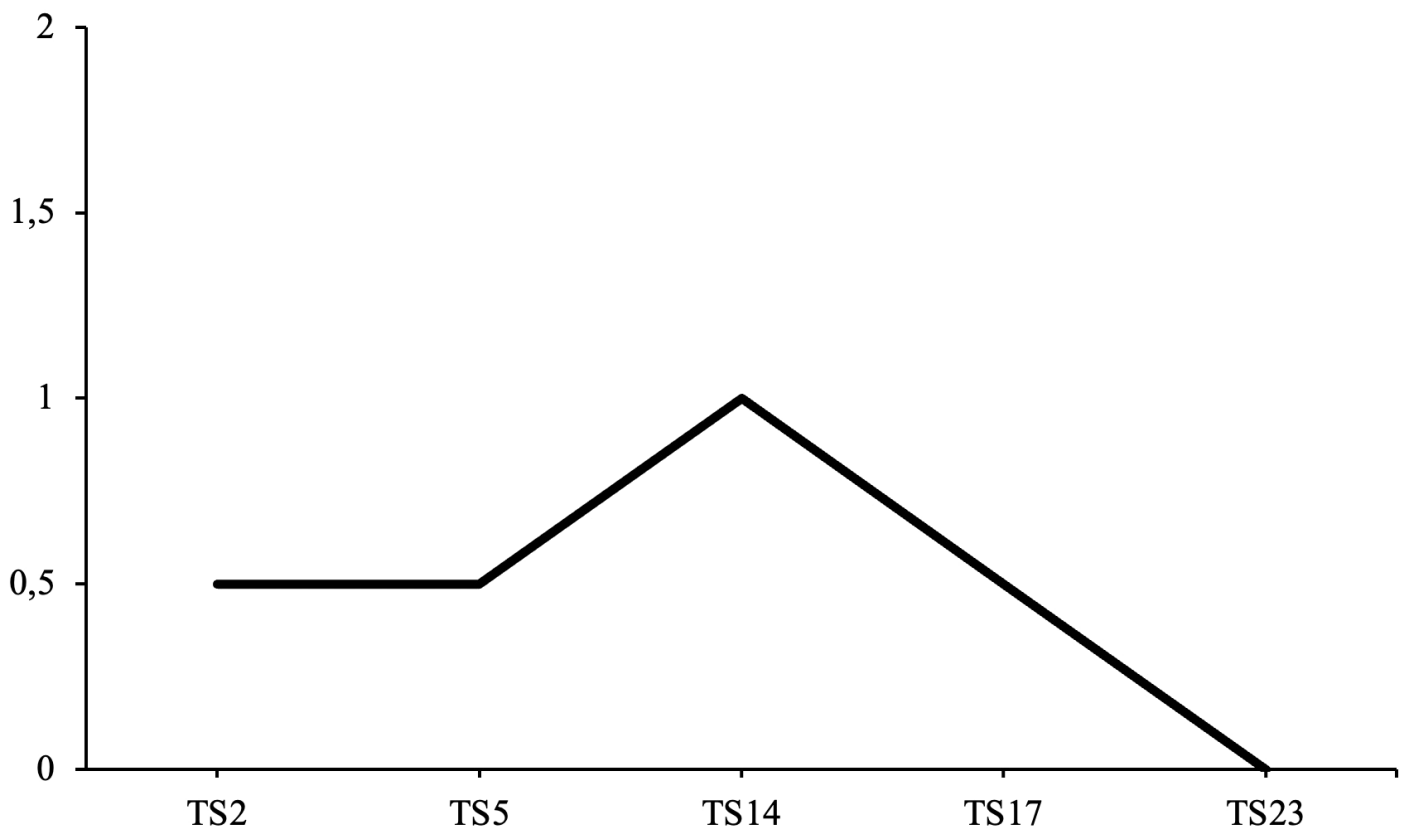
Average communion fulfillment scores over the course of treatment. TS, therapy session.

All average agency coding scores for all narratives from the therapy transcripts are illustrated in Figure 5. The first hypothesis predicted that the patient’s narratives would increase in agency over the course of psychotherapy (H1).

At the beginning of therapy during the second session, the patient’s agency is moderate, with a score of 1.88. These narratives portray the protagonist, to a certain degree, at the mercy of external powers and circumstances. During the second session, the patient describes her past romantic relationships. Before being in a relationship with her ex-partner, she had engaged in sexual intercourse with several men. She describes suffering from ambiguous thoughts concerning her experiences, as she felt she needed to catch up on lost time, but also suffered from feelings of being “cheap” or “used.” She describes the following impression she had of herself:

*Somehow, I sometimes had the feeling that I got [intimate] with someone too quickly or that I was too easy to have […].*

Once she had started dating her former partner, she had told him in a conversation about her past romantic experiences, which he had strongly disapproved of, which led to her criticizing herself:

*[…] And he [said to me], "yes, when I saw you, I did not think you were like that". And I thought that was pretty harsh. So, I know that I was also hurt by it, but I believed it; I took it that way, that it was something negative and something that was not appropriate, even though I knew that so many people actually did it.*

Instead of gaining agency by having actively sought out intimate relationships, the opposite occurs. The patient not only questions her own decisions and needs, but she does not describe herself as someone who wanted to have intimate experiences with others but instead was someone “easy to have.” She devalues her own sexual needs and allows her ex-partner to persuade her that her past behavior was inappropriate, even though she states that she believes others have the right to do so. The patient’s narratives do not include an agentic description of her seeking out intimate relationships or standing up for herself and her needs. Instead, she portrays herself as someone swayed by others’ preconceptions and accusations by criticizing herself for past desires. She does not consider herself in control of her experiences. Instead, she describes herself at the mercy of her needs which she simultaneously condemns due to the prejudices of her ex-partner. Despite her depicting experiences from the past, these narratives also lack insight, in which the patient corrects her past views and regains agency while narrating these events during psychotherapy.

During the fifth session, the patient’s agency decreases to a score of 1.29, displaying her as someone who is largely at the mercy of circumstances and that her experiences are primarily in the hands of external powers. The patient reports having issues in a new relationship, in which she has ambiguous feelings towards the man she is dating, as he is also interested in another woman. She describes having the desire to distance herself from him but being unable to do so, and that all her thoughts and emotions are circling the man she is dating, whereas she is unable to act or make a decision to influence her current relationship:

*I have noticed that I somehow do not get the distance in [..] and that my thoughts revolve around it, and I find it a bit of a shame because I somehow make myself so dependent on the reaction of the other person instead of looking out for myself [..], but all my thoughts always revolve around what he is thinking about, what he is feeling at the moment and I find that a bit of a shame that I am so fixated on that.*

The patient spends the therapy session talking about how she is unable to control her thoughts as she is so emotionally involved with her current partner. She describes herself, including her thoughts and emotions, as primarily controlled by his actions and decisions. Despite wanting to enjoy her time alone or with friends, she is unable to distance herself from the situation or specify what she expects from a relationship. She, therefore, depicts herself as a protagonist with a low agency, as she is at the mercy of his actions and her thoughts.

During the second third of therapy, the patient’s agency increases drastically to a moderately high score of 2.88 at session 14. This illustrates that the patient, to a certain degree, describes herself as someone who is able to affect her own life, initiate change and achieve some degree of control throughout her experiences. The patient reports going on holiday alone and getting to know others abroad. She also points out that she was not interested in having romantic encounters but instead decided to seek platonic friendships, with which she was content. She also displays a degree of insight by expressing her struggles with loneliness while traveling by herself:

*[…] the first evening I was […] restless, I noticed that I did not feel good at all, that [something] was missing, and I noticed [..] that even though traveling alone is actually nice [I realized] that I always need someone to talk to or do something with, because [..] it made me so restless [..] being all on your own, so alone.*

The patient not only acknowledges that she enjoys traveling alone but that she simultaneously feels a high degree of loneliness and restlessness when not having someone to talk to or spend time with. She describes herself as being highly agentic by traveling on her own but also describes being at the mercy of the need to be with others. Therefore, the theme of agency is slightly thwarted by inner struggles with needing others to feel at peace while also trying to be highly agentic by traveling alone. However, the patient also expresses insight into these inner struggles, with which future actions may be influenced, displaying a moderately high agency score.

Furthermore, the patient mentions that she has had contact with her ex-partner and has the impression that she can see more clearly that the breakup was necessary for her, as she felt suppressed by her ex-partner. She claims that now with more distance, she is able to understand that during the relationship, she did not seek out her own needs and did not act in her interest:

*[…] and now, with such a distance, we can see more clearly that we were simply too different and somehow wanted too many different things and that it just could not work out. And I was also so deep in it; you hold on so tightly to what you find beautiful that you do not see the other [sides of the relationship].*

The patient, therefore, experienced an insightful moment in which she acknowledged that she was not agentic during the relationship. With this insight, seeking out different relationships can be seen as a goal-oriented action in the future, resulting in an overall increase in agency.

At session 17, nearing the end of treatment, the patient’s agency score slightly decreases but remains moderately high, with a score of 2.67. The narratives of this session portray the protagonist as able to affect situations or initiate change to some degree. In one of the narratives, the patient breaks off a relationship in which she realizes that the man she is dating does not want to start a serious relationship. In this narrative, she portrays herself as highly agentic by distancing herself from a relationship in which her needs are not being met:

*I really want to stay on my own for now and not start [dating] anyone else because I want to concentrate on myself and see that everything is fine with myself first [..] what fulfills me, regardless of the fact whether there is a man with me now […].*

However, the patient depicts her struggles with her sexual desires and needs. In the second narrative, she illustrates her inner struggles with the topic, asking herself whether her upbringing is at fault for her being such a “needy” person. She, however, states that she has ambiguous feelings. She reports that having intimate experiences with others “just happens” but that she is a grown-up woman and can make her own decisions. She is, however, ashamed of seeking out intimate relationships, especially when first stating she wants to be on her own now. Therefore, in this narrative, she is neither highly agentic nor completely at the mercy of external powers; instead, these narratives illustrate a general struggle with the theme of agency.

Towards the end of therapy at session 23, the patient’s agency score increases slightly to a score of 3, reaching its peak since the beginning of therapy, portraying her as agentic and able to initiate change. In this session, her narratives evolve around an argument with her ex-partner, in which he insults her for having had intimate relationships with others since their breakup. The patient reports that she understands why he is upset but does not view her behavior as inappropriate, especially as she was not in a relationship with him. She also points out that when they were still together, she would always justify her actions to calm him down. She states that she can still feel the urge to justify her actions but is now aware that she is not in the wrong. Furthermore, she is determined to change her previous behavior by trying to create distance between them. She further reports that she directly told him that she would not tolerate his behavior toward her:

*[…] last time I said, "if you apologize, we can meet, but you were very hurtful, and if you do not apologize, we will not [meet up again]" […].*

The patient illustrates not only insight but stands up for herself in this situation and is determined to initiate change when it comes to her previous behavior, depicting herself as a protagonist that is in control of the situation and is able to initiate change to some degree.

The first hypothesis was partially supported, as agency increased over the course of psychotherapy, except for decreases in sessions 5 and 17 (H1).

#### 3.1.2. Communion fulfillment

**Figure 6:**
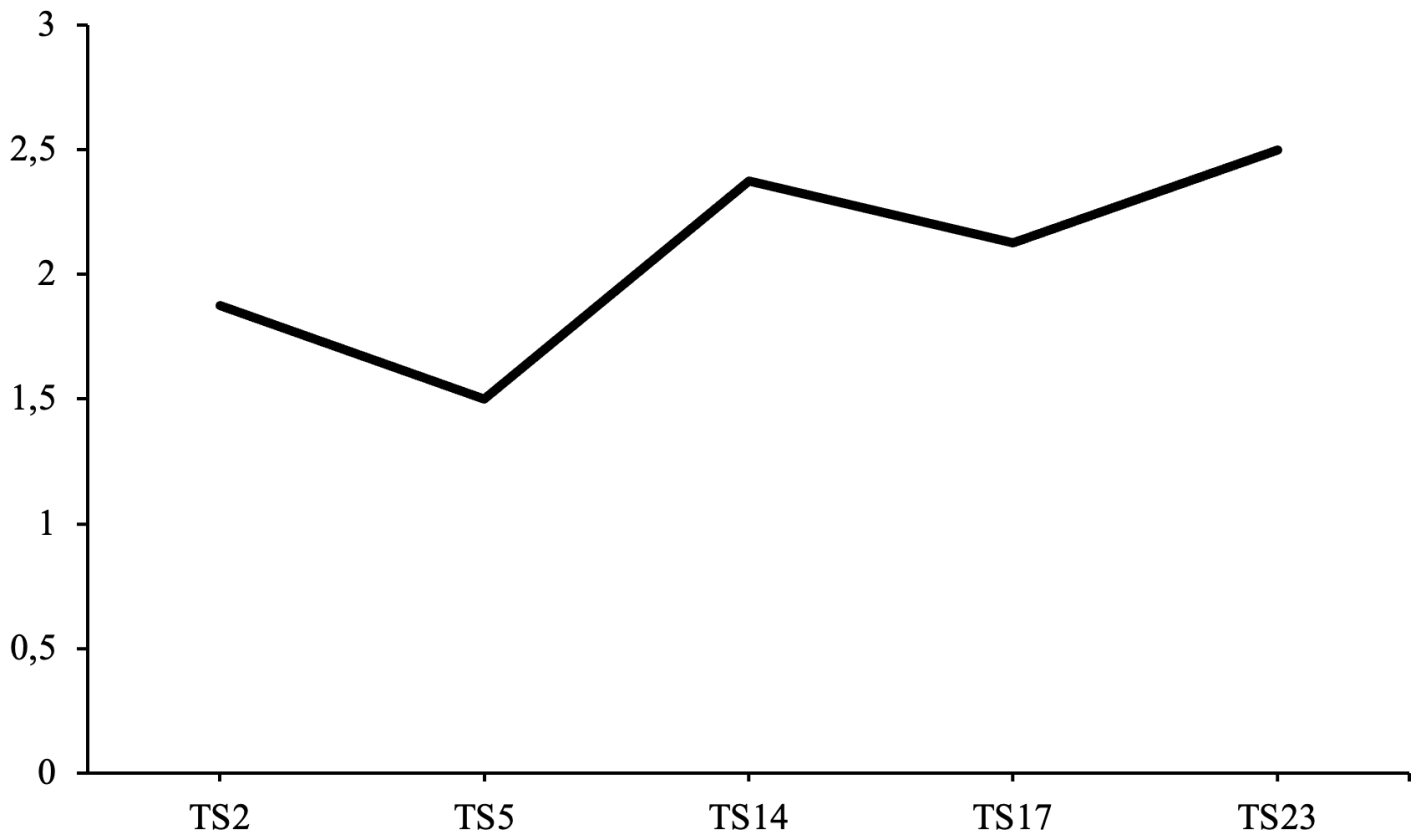
Average coherence scores over the course of treatment. TS, therapy session.

All communion fulfillment scores over the course of therapy are illustrated in Figure 6. The second hypothesis predicted that communion fulfillment would increase over the course of psychotherapy (H2).

At the beginning of therapy during the second session, the patient’s communion fulfillment is moderately low, with a score of 0.5. The patient depicts experiences in which she is either ashamed of her own needs or in which she feels disapproved of or devalued:

*[…] somehow, I had the feeling that I could be replaced at any time, so to speak. And it's all about sex […].*

Her communion fulfillment score remains stable at a score of 0.5 during the fifth therapy session, in which she does not feel that her expectations of a relationship are being. She reports being unable to satisfy her needs with her current partner; moreover, she reports being unable to move on and is fixated on him:

*[…] since then, he has been distant. […] instead of seeing for myself what is good for me at the moment and meeting up with friends, [..] all my thoughts are always [revolving around him], about what he is thinking, what he is feeling at the moment. And I find it a bit of a shame that I'm so fixated on that.*

Towards the second third of the patient’s treatment, her communion fulfillment reaches a moderate score of 1 in the 14th session. While traveling abroad, the patient meets new people and seeks conversations with others. However, she also describes herself as restless and lonely when she is on her own:

*[…] even though travelling alone is actually nice, [I noticed] that I always need someone to talk to or do something with, [it made me so restless] that you're somehow on your own, so alone.*

During this session, she also points out that she realizes that the relationship with her ex-partner was toxic and that she is better off since her breakup. She claims that she can now see more clearly that they were not seeking the same thing from a relationship, resulting in both being unhappy.

Towards the last third of therapy, the patient’s communion fulfillment score decreases again to 0.5 during the 17th session. The patient realizes that her current relationships are not fulfilling her needs and that she is unhappy with how others are treating her and that she would rather be on her own:

*[…] I really want to stay on my own for now and not start anything more with anyone because I want to concentrate on myself […].*

Before treatment termination, the patient does not experience any communion fulfillment, therefore receiving a score of 0 during the 23rd therapy session. Over the course of therapy, after having phases of being in contact with her ex-partner, the patient realizes in an argument she had the previous day that her ex-partner will only keep on devaluing her and that she does not want to put up with it anymore. She realizes that her past and current relationship with him does not fulfill her communal needs in any way:

*[…] and then I said that I didn't think it was good for us to keep seeing each other because I couldn't see a way back into the relationship […].*

Despite communion fulfillment increasing slightly towards the middle third of treatment, the patient’s communion fulfillment score had an overall negative trend. Therefore, these results do not support the second hypothesis (H2).

#### 3.1.3. Coherence

**Figure 7:**
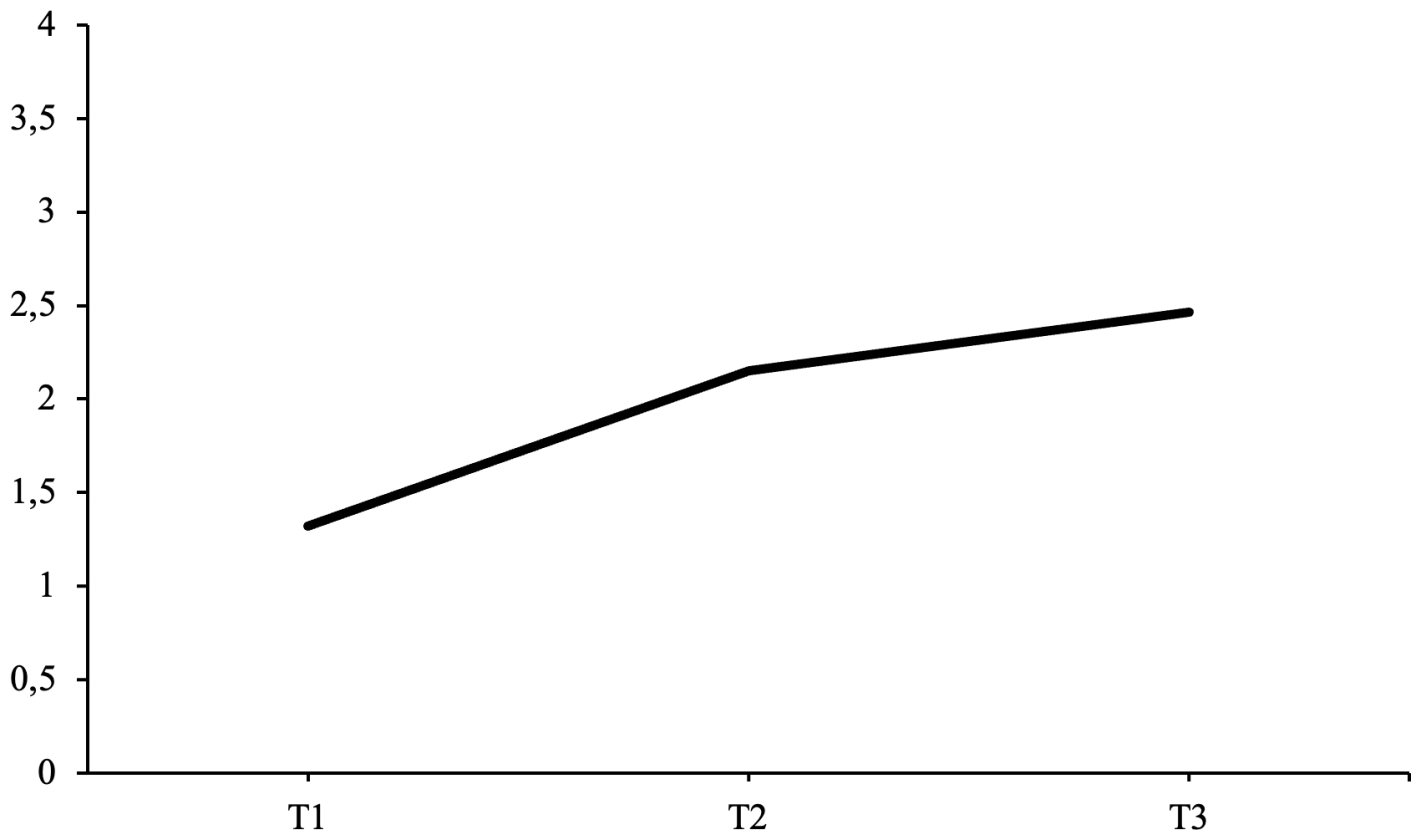
Average life story agency scores at each interview time point. T1, pre-interview; T2, post-interview; T3, follow-up interview.

All average coherence scores over the course of therapy are illustrated in Figure 7. The third hypothesis predicted that the overall coherence of the patient’s narratives would increase over the course of psychotherapy (H3).

The patient’s narratives are moderately high in coherence at the beginning of therapy, with a score of 1.88 in the second session. When describing her past romantic experiences and her conversation with her ex-partner, she, to a certain degree, orients the listener into the story, and one can identify the structure or the temporal sequence of all incidents. However, she only uses a small amount of affective language to describe how she felt during these experiences, but not why the story is important to tell. The two narratives also lack a high degree of integration of the story into her self-identity and are not linked to previous autobiographical experiences. She merely reports that she felt disappointed about others and herself.

The patient’s overall coherence score decreases to 1.50 at session five. Orientation and structure remain moderately high, allowing the reader to identify most major characters and locations as well as most of the temporal sequences of events; however, one of the narratives includes a low amount of affective language, whereas the other lacks both the use affective language and integration to a high degree. It does not become clear why these experiences are important for the patient to tell or how these make her feel. One of the narratives is also not integrated into her self-identity, as the patient merely portrays her struggle with her current dating life.

At the second third of treatment, overall coherence largely increases to a score of 2.38 at session 14. The coherence scores of both narratives range from moderately high to high on all four dimensions. The patient’s depiction of her traveling alone scores highly on three of four coherence dimensions. The structure dimension is the only one in which the patient only scores moderately high, as the overall sequence of events becomes unclear in certain passages. The patient’s elaboration on having a different view on her past relationship scores moderately high on all four dimensions.

In session 17, overall coherence slightly decreases to a score of 2.13. The patient’s first narrative evolving around wanting to be alone includes a high degree of integration; however, there is a lack of affective language, and it does not become clear to the reader how the patient felt during that specific event. The second narrative in which the patient depicts struggling with current intimate relationships and identifying her own needs, orientation but especially structure decrease, and there is a lack of affective language, whereas integration remains high.

At session 23, the patient’s overall coherence score increases and reaches its highest rating before treatment termination, with a score of 2.50. Both narratives include a moderately high to high range of coherence on all four dimensions. However, the overall integration of both narratives has slightly decreased compared to the 17th session, from a score of 3 to 2.

The third hypothesis was partially supported, as overall coherence increased over the course of therapy, with the exception of decreases in sessions 5 and 17 (H3).

### 3.2. Life stories

#### 3.2.1. Agency

**Figure 8:**
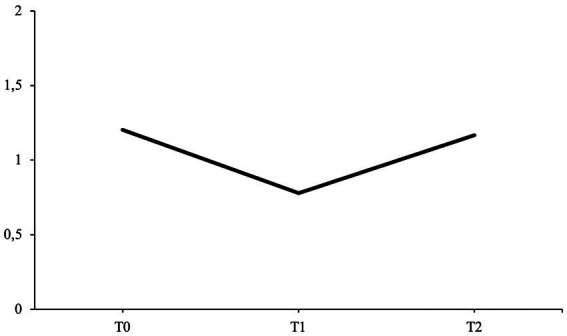
Average life story communion fulfillment scores at each interview time point. T1, pre-interview; T2, post-interview; T3, follow-up interview.

All average agency scores at each interview time point are illustrated in Figure 8. The fourth hypothesis predicted that the average agency scores at T2 and T3 would be higher when compared to the average agency score at T1 (H4).

During the pre-interview (T1), the patient’s average agency score starts at 1.32, illustrating herself mostly at the mercy of circumstances in her life experiences. She starts by describing her birth complications and following health issues, due to which she experienced a slower progression while growing up compared to other children. She also narrates about her struggles connecting with others and experiencing bullying during middle and high school, in which she portrays herself at the mercy of the actions of others and specific circumstances. She then describes a turning point in her life, in which she decided to initiate change and travel abroad for a year. She gained self-esteem through her year abroad and perceived herself as highly agentic.

During the post-interview (T2), the patient’s average agency score increased to 2.15. She again narrates about her birth complications, health issues, bullying experiences, and year abroad. However, the patient also narrates how she experienced the break-up with her ex-partner and why she decided to start therapy. The new narratives included in her life story portray her as agentic, as she includes many points of self-insight that have brought her to the point in life she is now, due to which she has changed her behavior and attitudes. She reports that these experiences have helped her grow as a person:

*[…] but I went through it all because I always hoped that somehow the trust would grow, and the relationship would become easier […]*

*[…] I let him do a lot to me, but then I realized that it was just a relief later on. And I started therapy directly afterwards [..] [in order] to learn to distance myself and to say no. And to learn not to feel guilty anymore and that I am very important and that I can't let something like that happen to me. So, this experience was very important for me.*

At the follow-up interview (T3), the patient’s average agency score slightly increases again, reaching a final score of 2.46. Her life story again includes her depiction of her birth complications, health issues, and bullying experiences. Her depiction lacks a sense of agency, as the portrayal of herself remains at the mercy of past circumstances. She also narrates about her year abroad, in which she portrays herself as highly agentic. She also mentions the comparison with her sister, depicting this relationship as a reason for wanting to travel abroad, initiate change, and distance herself from her previous friend group and the stigmata of being the sister that is not as capable of being self-assured or agentic. She also narrates about a past breakup due to feeling that the relationship was not fulfilling her communal needs. She finishes the interview by narrating again about ending the relationship with her ex-partner:

*[…] but at the end of the day it was an important experience for me because it was like a liberating blow, and I found myself again […]*

Compared to the post-interview, the narratives of the follow-up interview include more passages of self-insight and reasoning for her past decisions, depicting an overall higher score for agency.

The results support the fourth hypothesis, as agency scores at T2 and T3 are higher compared to the baseline score at T1 (H4).

#### 3.2.2. Communion fulfillment

**Figure 9:**
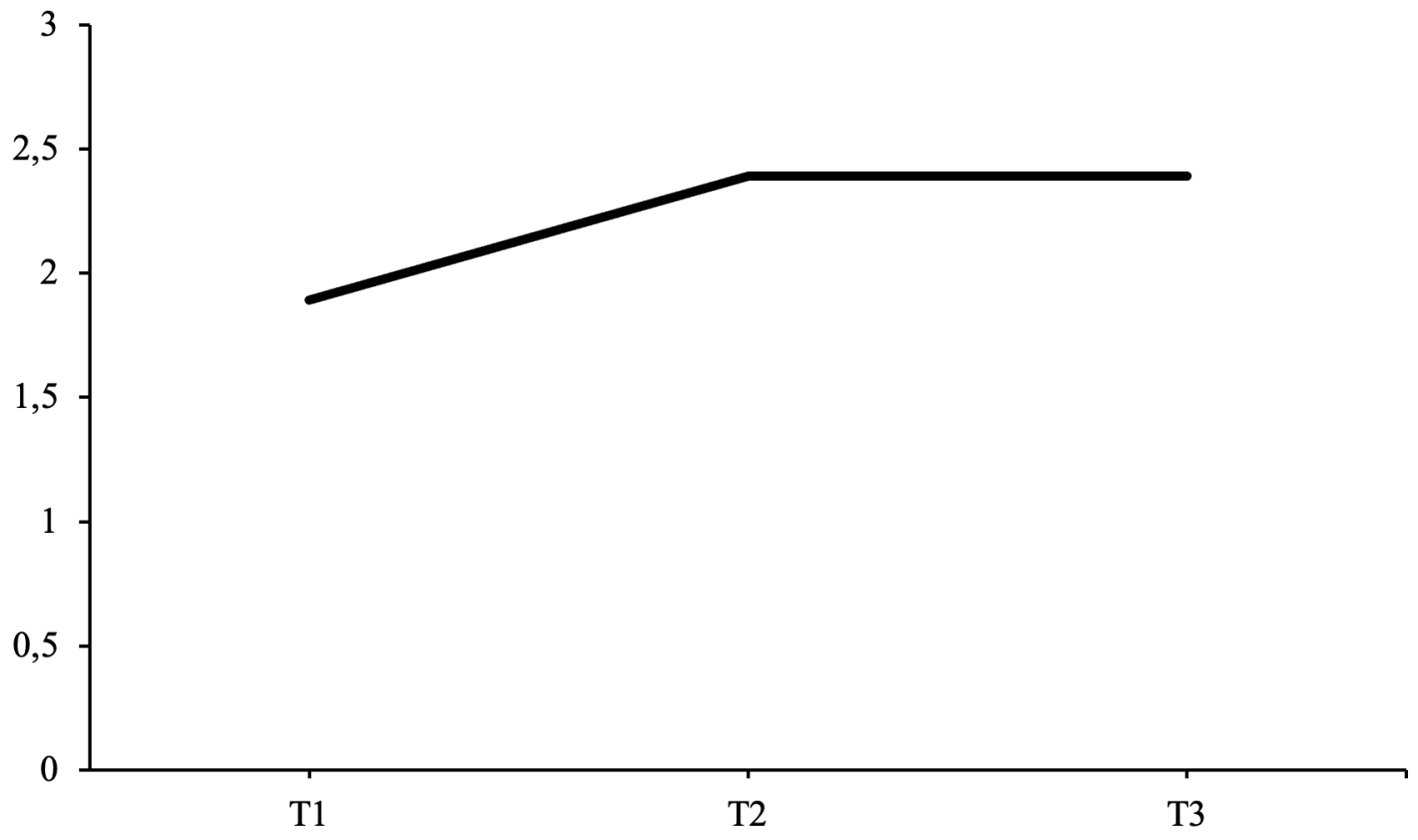
Average life story coherence scores at each interview time point. T1, pre-interview; T2, post-interview; T3, follow-up interview.

The patient’s average communion fulfillment scores for each interview time point are presented in Figure 9. The fifth hypothesis predicted that communion fulfillment scores at T2 and T3 would be higher when compared to the communion fulfillment score at T1 (H5).

From the pre-interview (T1), four out of seven narratives were coded for communion fulfillment; from the post-interview (T2), six out of seven narratives were coded; and from the follow-up interview (T3), six out of seven received a communion fulfillment rating.

In the pre-interview (T1), the patient has a moderate communion fulfillment score of 1.20. As a child, she describes herself as being afraid of seeking contact and playing with other children and that she would often feel ashamed when picked on. Due to being shy, she also experienced difficulties standing up for herself and befriending new people. Over the course of her school career, during which the patient experienced phases in which classmates were bullying her, she also realized that she was still perceived by her friends and family as shy and inhibited even though her self-perception had changed, causing her to resent her current relationships. This was also one reason for her year abroad, where she describes having had great friendships and feeling loved by others.

In the post-interview (T2), the patient’s communion fulfillment score decreases to a moderately low score of 0.78. During this interview, she again narrates her previous struggles in past relationships, as in the pre-interview. However, the patient also includes more narratives in which these struggles become more prominent, for example, always being compared with her sister and her failed relationship with her ex-partner, resulting in her seeking treatment. She illustrates herself as becoming aware of how unsatisfied she is with her past behavior in her previous relationship and how she sacrificed friendships and her own communal needs for her ex-partner:

*I tried to do everything for him to keep this relationship going well and then I almost gave up on myself, I gave up male friendships for him because he didn't want that and I did less with friends, so I gave up a lot for him [..] and the experience of the break-up devastated me [..] but in the end it was an important event for me [..] it's like a liberation blow and I found myself again.*

At the follow-up interview (T3), the average communion fulfillment score for the patient’s life stories increases to a moderate score of 1.17, almost reaching the same score as the initial starting score of the pre-interview. During this interview, the patient narrates about negative experiences while in school and being compared to her sister. She also mentions the break-up with her ex-partner. However, she also elaborates on friendships she made during her year abroad, having a beneficial working relationship with her therapist and founding a sports club with friends.

These results do not support the fifth hypothesis, as both scores at T2 and T3 are lower when compared to the communion fulfillment score at T1 (H5).

#### 3.2.3. Coherence

The patient’s average coherence scores at each interview time point are illustrated in Figure 9. The sixth hypothesis predicted that overall coherence scores at T2 and T3 would be higher compared to the overall coherence baseline score at T1 (H6).

Before the beginning of treatment (T1), the patient has a moderate coherence score of 1.89. The life story narratives score high on orientation and structure, allowing the reader to identify the main characters and sequence of events. However, many narratives lack affectual language or integration, especially in which she depicts herself struggling with certain circumstances such as health issues and bullying experiences.

In the post-interview (T2), the overall coherence of the patient’s life story narratives increases, reaching an average score of 2.39. Not only do the scores for orientation and structure increase, but the patient’s use of affectual language and especially integration largely increases as she integrates her autobiographical experiences into her self-identity. Integration became especially prominent in narratives regarding her break-up and reason for seeking treatment.

In the follow-up interview (T3), the patient’s overall coherence score remains stable, having the same overall score as in the post-interview. In comparison to the post-interview scores, orientation and structure scores further increased, and the use of affective language remained stable, whereas the integration score decreased. Most of the narratives that had been previously narrated during the post-interview had slightly decreased in overall coherence. In contrast, newly narrated life stories, such as founding a sports club and depicting an important therapy session, scored moderately high in coherence.

These results support the sixth hypothesis, as coherence scores are higher at T2 and T3 compared to the baseline score at T1 (H6).

## 4. Discussion

To the best of our knowledge, this is the first study to examine the development of the narrative identity of an AvPD patient in both narratives within STPP sessions and life stories before, after, and 6 months following treatment termination. It was predicted that agency, communion fulfillment, and coherence would increase over the course of psychotherapy. Moreover, it was hypothesized that the life stories at the post-and follow-up interviews would be higher in agency, communion fulfillment, and coherence compared to the pre-interview life story. The results partially supported these hypotheses. On the one hand, agency increased with a few declines over the course of psychotherapy and increased further 6 months following treatment termination. Comparatively, communion fulfillment began to increase slightly and decreased drastically towards the end of treatment. Six months following treatment termination, communion fulfillment had almost reached its previous starting point. Coherence, on the other hand, increased with a few decreases, similar to agency over the course of psychotherapy, and remained stable 6 months following treatment termination.

### 4.1. Increases in narrative agency and coherence are associated with successful psychotherapy outcome

As predicted, agency scores increased over the course of psychotherapy in the patient’s narratives evolving around romantic interpersonal relationships and increased further after treatment termination. These findings align with previous studies that have also found agency to increase in life stories after psychodynamic psychotherapy treatment of BPD patients (35) or in narratives regarding psychotherapy of patients with a variety of diagnoses throughout a 12-week treatment program (22).

Due to a decrease in overall symptom severity and a decrease in BDI-II and BSI scores after treatment and 6 months following treatment termination, it can be established that the case study had a successful treatment outcome. Despite agency being considered a major motivational thematic cluster in personal narratives and life stories (20), the construction of highly agentic narratives does not necessarily result in an individual acting agentic. However, an increase in agency within personal narratives may reflect the manifestation of change within behavior in everyday life. Due to the increase in agency within the therapy narratives as well as life stories and a decrease in depressive symptoms, it may be presumed that the patient also acted more agentic within her everyday life. This assumption would conclude that an increase in agency may result in an improvement in mental health, which would align with previous theories, suggesting agency is a key factor for positive psychological functioning (22, 64, 67). Furthermore, an increase in agency in the patient’s life story remained consistent even after treatment termination, suggesting that psychotherapy may have long-lasting effects on agency development.

It should also be pointed out that the patient displayed decreases in sessions 5 and 17, in which she overall struggled to gain control over situations and felt partly at the mercy of circumstances due to an inability to influence her interpersonal relationships. These results indicate that working through narratives in which the individual may struggle with gaining agency does not occur in a linear pattern. Instead, working through these themes consists of “ups and downs” which is found to be common in psychotherapy treatment (68) and is thus a good representation of the development of agency throughout psychotherapy.

As predicted, overall coherence increased over the course of psychotherapy, similarly to agency and unexpectedly remained stable after treatment termination. These findings align with those of studies conducted with patients diagnosed with posttraumatic stress disorder (43), schizophrenia (44, 45) and BPD (46) which have also concluded that life stories and personal narratives are more coherent after psychotherapy. However, it does not align with Adler’s previous findings (22), as it was concluded that coherence did not increase throughout a 12-week psychotherapy treatment. However, these findings were based on narratives written by participants after each session and revolved around experiences regarding psychotherapy. Therefore, these narratives demonstrate a more filtered form of narrative, which can not be compared to narratives examined within psychotherapy sessions. Furthermore, the present study’s findings contradict the assumption that coherence in past accounts increases over time, whereas narratives regarding present accounts remain unchanged over the course of psychotherapy (22). Narratives used for coding mainly regarded present experiences at the time. This suggests that time may not be the only factor in developing higher coherence. Instead, these findings indicate that narrating events more coherently portrays better coping with experiences and improvements in overall mental health. Due to the decrease in symptom severity over the course of psychotherapy, results may demonstrate a relation between higher well-being and coherence which would align with previous findings (22, 64). As mentioned above, the decreases in sessions 5 and 17 may demonstrate the non-linear pattern of working through “ups and downs” commonly found in psychotherapy treatment (68).

Due to having a similar development as agency over the course of treatment, it may be presumed that coherence and agency are highly associated. The ability to narrate past experiences and one’s life story in a highly coherent manner expresses how well an experience has been dealt with and provides a sense of meaning (23–25). Furthermore, an individual that narrates with a high degree of agency is also said to experience a sense of meaning and purpose (9, 20). Therefore, agency and coherence are associated with the utilization of meaning-making; thus, regaining agency and, with that, a sense of control over circumstances might simultaneously increase the ability to narrate more coherently.

### 4.2. Narratives low in communion fulfillment may reflect awareness on maladaptive relationship patterns

Unexpectedly, communion fulfillment only increased slightly towards the middle third of psychotherapy but was non-existent by treatment termination. This finding is surprising, as communion fulfillment has been associated with high mental well-being (22, 69), due to which it was expected that over the course of successful psychotherapy treatment, communion fulfillment would increase. Furthermore, previous findings have not reported an overall decline in communion fulfillment after psychotherapy but instead suggest that communion fulfillment remains unchanged (28).

A possible reason for decreased communion fulfillment in this case study could be the form of treatment. Psychodynamic psychotherapy focuses on exploring past relationship patterns and working through central conflicts within interpersonal experiences (52, 53). Over the course of treatment, the patient became aware of how unfulfilling her relationships are, as her relationship needs were unmet, and her feelings were not reciprocated. In the last third of treatment, the patient states that she might need time to focus on herself and develop a better understanding of what she seeks in a healthy relationship. It may therefore be suggested that the first step in this patient’s case study is gaining agency. An increase in agency allows the patient to become aware that she is not at the mercy of the actions of others regarding interpersonal relationships but that she may also make decisions and seek what is most fulfilling for her. The second step would be for the patient to form and maintain healthier and more satisfying relationships. Furthermore, it should be pointed out that the patient underwent short-term treatment. Therefore, when undergoing long-term treatment, the patient’s communion fulfillment may become balanced with her level of agency over the course of psychotherapy.

The patient’s communion fulfillment score had only increased at the six-month follow-up interview, whilst almost reaching the initial starting score This may suggest that the following two factors have occurred: (1) the psychodynamic setting encourages patients to critically evaluate their current relationships (52, 53). However, this effect may have decreased over time once treatment was terminated, suggesting that a critical viewpoint may either be only present when undergoing treatment or may have long-lasting effects after long-term treatment, as has been suggested by previous studies comparing short-term vs. long-term psychodynamic psychotherapy treatment (2, 70) the patient may have also started seeking out more fulfilling interpersonal relationships, as she had become aware of what relationships she would want to pursue in order to feel fulfilled in her interpersonal experiences. In her life story at the follow-up interview, the patient narrated about past and present positive relationships, which had not been mentioned in the pre-and post-interview. This would underline that she became aware of fulfilling relationships in the past and present. These results would align with the theories presented by Dimaggio et al. (71), namely that self-development and self-reflection precede the development of relationships with others.

### 4.3. Limitations of the present study

The current study has several limitations that must be taken into consideration when interpreting these results. Firstly, due to this being a case study, generalizability is limited. However, the examination of case studies can be beneficial to decipher future research possibilities and allow for a more in-depth investigation (72). The lack of blinding of the master coder may also be listed as a limitation of the present study. However, the high inter-rater reliability established by the reliability coder could be understood as a confirmation of a sufficient objective coding provided by the master coder. A possible limitation is only examining narratives revolving around romantic relationships. This is only one form of relationship issue that the patient suffered from, as she also reported struggling at work and within friendships. However, narratives regarding interpersonal romantic relationships allow us to examine the development of narrative identity on a very specific scale (73), as these may portray the overall general behavior in other interpersonal relationships, which is a common concept in psychodynamic psychotherapy (52, 53).

### 4.4. Suggestions for future research

Despite the current case study including a lack of generalizability, this case study, especially the study design, may be used as inspiration for future research. Further studies should inspect the development of narrative identity not only before and after psychotherapy treatment but also examine narratives within psychotherapy sessions. Multiple research studies have linked communication fulfillment and coherence to high mental well-being. However, findings of previous studies, which have reviewed the development of communion fulfillment and coherence in patients receiving psychotherapy, are inconsistent with narrative identity theory. Furthermore, it should be noted that only a few studies have specifically examined changes in agency, communion fulfillment, and coherence. Moreover, these studies only compare scores before and after psychotherapy or use narratives written by patients after psychotherapy sessions. Narratives within psychotherapy should be examined in order to identify crucial moments of narrative identity development and create a better understanding of possible influencing factors. We suggest emphasizing the importance of examining narratives within psychotherapy treatment by the distinction between the following two settings: interview and therapeutic setting. During an interview, the participant is asked to narrate their life story without interruption. This may result in the participant reporting a life story with as much detail as possible. In comparison, therapy sessions include a dialog between the patient and therapist. Moreover, patients may use narratives to present their psychological distress and, therefore, present themselves with more vulnerability. Psychotherapy aims to work through important narratives together, resulting in a different narration form than in an interview setting. Therefore, the processes leading to changes in narratives and narrative identity may be more observable when examining narratives within psychotherapy. Due to the lack of research on narratives within therapy sessions, further studies are vital to uncovering differences in narration due to the specific setting in which narratives are reported.

Furthermore, it should be examined whether changes in narrative identity truly result from the intense working process within psychotherapy or whether changes occur naturally over time, regardless of whether receiving treatment. This may be investigated by using a control group of patients who only undergo the interview assessments, without receiving treatment in the given time frame. This may uncover whether changes in narrative identity occur without treatment and, if so, whether these may be pinpointed to merely repeated narration.

Studies should also consider comparing different treatment forms of psychotherapy and their effects on changes in narrative identity. This study concluded that STPP focuses on conflicts within interpersonal relationships, encouraging patients to become aware of their unhealthy relationship patterns and whether they feel fulfilled within their current relationships. Therefore, it should be examined whether other forms of psychotherapy, such as less conflict-focused treatments like cognitive behavioral therapy, or treatments with a longer duration, such as psychoanalysis, may have the same impact on communion fulfillment.

Moreover, it should be investigated whether these findings can be generalized and whether these outcomes are characteristic of people suffering from AvPD. There is a lack of narrative identity research on other forms of PDs besides BPD. The importance of a narrative identity approach in the treatment of PDs has been stressed by Lind (74), as this allows for a more dimensional approach since narrative identity is considered a mechanism of change within psychotherapy treatment of moderate to severe PDs (75). Working with life stories and personal narratives emphasizes the importance of a patient’s unique life experiences and strengthening narrative identity within one’s life story may result in a healthy organization of personality (74). Furthermore, AvPD is considered a neglected PD. Due to its high prevalence, mortality rate, and subjective impairment, further narrative identity research, including a larger sample size, must be conducted in order to identify effective treatment forms for AvPD, which include a narrative turn (74).

### 4.5. Conclusion

This case study extends the findings of already existing literature on narrative identity development in relation to personality pathology and the impact of psychotherapy. Results illustrate that agency and coherence increase over the course of STPP and that agency increases 6 months after treatment termination. Regarding communion fulfillment, decreases were identified by the end of STPP and an increase 6 months following treatment termination. These results demonstrate how narrative identity of patients with a AvPD diagnosis may change throughout and after STPP. Moreover, the form of psychotherapy and its duration may impact how communion fulfillment develops throughout treatment.

Despite being a case study, through which generalizability is limited, to the best of our knowledge, this is the first study to examine narrative identity not only in life stories before, after, and 6 months following psychotherapy but also in narratives within psychotherapy sessions. These findings, therefore, emphasize the need for further exploration of psychotherapy narratives within the research field of narrative identity.

Future examination of the narrative identity development of individuals with AvPD should be conducted to fully understand the disturbances and changes in narratives by AvPD patients, especially since AvPD has largely been neglected by previous research. Further investigation dealing with these research gaps is vital as a narrative approach has been considered crucial for improving psychotherapy treatment of individuals with PD.

## Data availability statement

The original contributions presented in the study are included in the article/supplementary material, further inquiries can be directed to the corresponding author.

## Ethics statement

Written informed consent was obtained from the individual for the publication of any potentially identifiable images or data included in this article.

## Author contributions

DF contributed to conception and design of the study and organized the database. AT performed the qualitative analysis and wrote the first draft of the manuscript. All authors contributed to manuscript revision, read, and approved the submitted version.

## Conflict of interest

The authors declare that the research was conducted in the absence of any commercial or financial relationships that could be construed as a potential conflict of interest.

## Publisher’s note

All claims expressed in this article are solely those of the authors and do not necessarily represent those of their affiliated organizations, or those of the publisher, the editors and the reviewers. Any product that may be evaluated in this article, or claim that may be made by its manufacturer, is not guaranteed or endorsed by the publisher.
